# High-Performance Supercapacitors Using Compact Carbon Hydrogels Derived from Polybenzoxazine

**DOI:** 10.3390/gels10080509

**Published:** 2024-08-02

**Authors:** Shakila Parveen Asrafali, Thirukumaran Periyasamy, Jaewoong Lee

**Affiliations:** Department of Fiber System Engineering, Yeungnam University, 280 Daehak-ro, Gyeongbuk, Gyeong-san 38541, Republic of Korea

**Keywords:** polybenzoxazine, calcination, aerogel, porous structure, electrode materials

## Abstract

Polybenzoxazine (PBz) aerogels hold immense potential, but their conventional production methods raise environmental and safety concerns. This research addresses this gap by proposing an eco-friendly approach for synthesizing high-performance carbon derived from polybenzoxazine. The key innovation lies in using eugenol, ethylene diamine, and formaldehyde to create a polybenzoxazine precursor. This eliminates hazardous solvents by employing the safer dimethyl sulfoxide. An acidic catalyst plays a crucial role, not only in influencing the microstructure but also in strengthening the material’s backbone by promoting inter-chain connections. Notably, this method allows for ambient pressure drying, further enhancing its sustainability. The polybenzoxazine acts as a precursor to produce two different carbon materials. The carbon material produced from the calcination of PBz is denoted as PBZC, and the carbon material produced from the gelation and calcination of PBz is denoted as PBZGC. The structural characterization of these carbon materials was analyzed through different techniques, such as XRD, Raman, XPS, and BET analyses. BET analysis showed increased surface of 843 m^2^ g^−1^ for the carbon derived from the gelation method (PBZGC). The electrochemical studies of PBZC and PBZGC imply that a well-defined morphology, along with suitable porosity, paves the way for increased conductivity of the materials when used as electrodes for supercapacitors. This research paves the way for utilizing heteroatom-doped, polybenzoxazine aerogel-derived carbon as a sustainable and high-performing alternative to traditional carbon materials in energy storage devices.

## 1. Introduction

The ongoing energy crisis is driving the urgent need for clean and sustainable energy sources like solar, wind, and hydrogen power. However, integrating these renewables into the existing power grid requires robust energy storage solutions. Among the various contenders—lithium-ion batteries, zinc-ion batteries, and supercapacitors—supercapacitors hold immense promise due to their unique properties. Supercapacitors boast exceptional power density, allowing for rapid charging and discharging [1,2]. They also exhibit remarkable cycling stability, lasting through numerous charge/discharge cycles without significant degradation. Additionally, supercapacitors prioritize safety, making them ideal for applications where battery flammability poses a risk. These advantages have propelled supercapacitors into widespread use, powering everything from portable electronics to automotive systems and even new energy plants. The heart of a supercapacitor’s performance lies in its electrode materials [3,4,5].

Traditionally, carbon-based materials have dominated this space, with graphene emerging as a frontrunner due to its exceptional physical and chemical properties. However, graphene’s path to supercapacitor supremacy has not been without hurdles. A significant challenge lies in the strong attraction between graphene sheets, causing them to clump together. This aggregation hinders the flow of ions, essential for energy storage, and reduces the surface area available for interaction. In turn, this translates into a lower electric double-layer capacitance (EDLC), the mechanism by which supercapacitors store energy [6]. Additionally, graphene electrodes tend to have low packing density, hindering their ability to store energy efficiently within a limited volume. Finally, pure graphene lacks the necessary sites for pseudocapacitive reactions, which contribute significantly to energy density. Researchers are actively tackling these limitations to unlock the full potential of graphene-based supercapacitors. Strategies to prevent sheet aggregation, increase packing density, and introduce pseudocapacitive sites are at the forefront of this endeavor. By overcoming these challenges, graphene-based supercapacitors can revolutionize energy storage, paving the way for a future powered by efficient, reliable, and clean energy solutions [7,8,9].

Scientists are constantly innovating to improve the performance of graphene electrodes in supercapacitors. One key area of focus is packing density. Techniques like vacuum filtration and mechanical compression have shown promise in this regard, leading to more densely packed graphene and potentially improved volumetric performance. However, there is a catch: these methods can cause significant clumping of the graphene sheets. This clumping acts as a barrier to ions, the charged particles crucial for fast energy storage and release. This ultimately hinders the rate capacity, i.e., the device’s ability to deliver power quickly. To address the aggregation issue, various studies have explored the use of physical spacers between graphene layers. These spacers are intended to prevent the sheets from clumping together too tightly, allowing for better ion movement. Despite this, when aggregation becomes too pronounced, these spacers may fail to ensure adequate electrolyte penetration into the material. This results in a reduced effective specific surface area, limiting the energy storage capacity of the sample [10,11,12,13].

An alternative approach involves incorporating pseudocapacitive materials into the graphene structure. This strategy not only increases the packing density but also enhances the pseudocapacitive properties of graphene. Pseudocapacitive materials can significantly boost the energy density of graphene-based electrodes. However, this comes with trade-offs; the introduction of these materials can adversely affect the high-current charging and discharging performance of the graphene, ultimately shortening its service life. While these materials offer the potential for achieving ultra-high energy density, careful consideration of their impact on the overall performance and longevity of the electrode is crucial [14,15,16,17,18].

The pseudocapacitance is brought about by doping graphene with specific heteroatoms like boron, nitrogen, sulfur, and phosphorus. These elements significantly improve the material’s pseudocapacitive performance. Unlike battery-type pseudocapacitive materials, the Faradic reactions occurring between the doped functional groups and the electrolyte exhibit superior reversibility. This translates into a dramatic improvement in the cycle life of the graphene supercapacitors. Additionally, heteroatom doping can fine-tune the electronic transport properties and hydrophilicity of graphene. These modifications optimize ion transport and enhance the rate capability, leading to supercapacitors with exceptional performance across various metrics.

In essence, this approach leverages the unique properties of GO and strategic manipulation of the final structure to create a new generation of supercapacitor electrodes with superior performance, cyclability, and overall functionality [19,20,21,22].

This research builds upon previous advancements [23] by introducing a polymer gel-based carbon electrode with exceptional potential for energy storage applications. The key component of this electrode is a custom-designed benzoxazine monomer (Bzo) comprised of eugenol and ethylene diamine. This Bzo monomer forms the foundation for polybenzoxazine (PBz), a class of advanced resins boasting several advantages: Superior Stability—PBzs exhibit minimal water absorption and shrinkage during formation. Additionally, they undergo catalyst-free polymerization and retain their shape effectively. Tailored Functionality—the molecular structure of PBz can be strategically modified to suit specific applications. The Bzo monomer synthesis utilizes a Mannich condensation reaction, yielding a self-polymerizable structure upon heating. This process results in a PBz rich in hydroxyl (–OH) and amine (–NH_2_) groups. Two different polybenzoxazine-based carbon materials were produced. One through carbonization of PBz, namely PBZC, and the other through the gelation and carbonization of Pbz, namely PBZGC. To assess the performance of these polybenzoxazine-based carbon materials, a thorough analysis was conducted within a three-electrode system. Rigorous electrochemical evaluations were employed to determine their energy storage capacity and stability under various conditions. The results convincingly demonstrated the remarkable durability and efficiency of PBZC and PBZGC, positioning them as highly promising materials for energy-storage applications. The innovative composition and robust structural properties of these PBz-based carbon materials signify their immense potential for revolutionizing energy storage technology.

## 2. Efficient Synthesis of Ethylene Diamine Benzoxazine (EEd-Bzo) and Its Transformation into a Porous Carbon Material (PBZC)

### 2.1. Synthesis of EEd-Bzo Monomer

The synthesis of the EEd-Bzo monomer proceeded through Mannich condensation. The detailed procedure was as follows: a three-necked, round-bottom flask equipped with a magnetic stirrer and a reflux condenser served as the reaction vessel. The process began by dissolving paraformaldehyde (1.8 g, 0.06 m) in 20 mL of dimethyl sulfoxide (DMSO) at 50 °C with continuous stirring. Ethylene diamine (1.34 mL, 0.02 m) was then added dropwise into the reaction mixture. Simultaneously, a separate solution was prepared by dissolving eugenol (3.28 g, 0.02 m) in 10 mL of DMSO. Once the addition of ethylene diamine was complete, the eugenol solution was gradually added dropwise to the reaction mixture, while increasing the temperature up to 120 °C, and the reaction proceeded for 3 h at this temperature. The formation of pale-yellow solution indicated the completion of the reaction. After cooling to room temperature, the reaction solution was precipitated in 1 N NaOH and meticulously washed with distilled water to eliminate impurities, yielding the EEd-Bzo monomer. The final product was separated via filtration and dried under vacuum at 50 °C for 12 h to ensure the complete removal of moisture.

### 2.2. Conversion of EEd-Bzo Monomer to PBZC

**Scheme 1:**
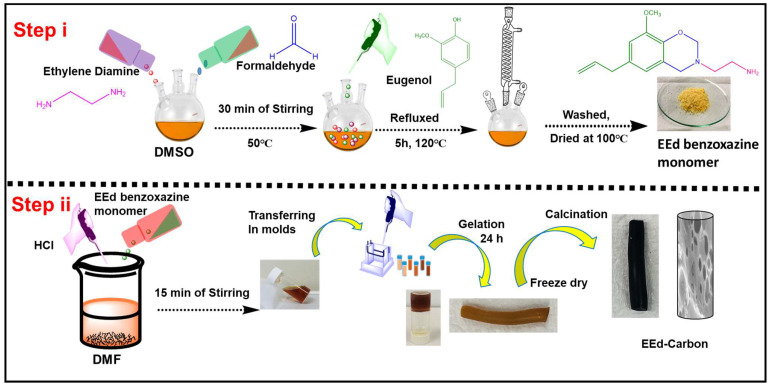
Schematic illustration showing the preparation process of EEd-Bzo monomer (Step i) and PBz-based carbon aerogel (Step ii).

The synthesized EEd-Bzo monomer underwent a stepwise thermal curing process. The benzoxazine monomer, EEd-Bzo, was subjected to progressively increasing temperatures of 100, 150, 200, and finally 250 °C, with each stage lasting for 2 h. This curing process transformed the EEd-Bzo monomer into a self-cured polybenzoxazine (PBz) network. Following the curing step, the poly (EEd-Bzo) was further processed by carbonization. This involved heating the material under a nitrogen atmosphere to 600 °C for 5 h, with a controlled heating rate of 1 °C/min. The resulting carbonized material was then thoroughly mixed with an aqueous KOH solution, with subsequent removal of water from the mixture through evaporation at 120 °C. The final stage involved the activation of the carbonized material, where the mixture was heated up to 800 °C (with a heating rate of 5 °C/min and holding at this temperature for 3 h) in a tubular furnace under continuous nitrogen flow. The final product obtained from this step was the desired PBZC material. Scheme 1 provides a visual illustration of the entire synthesis and activation process for PBZC. This method offers a reliable approach for synthesizing a high-quality benzoxazine monomer and its subsequent transformation into a well-defined carbonized and activated material, making it suitable for diverse applications. The meticulous control of temperature and reaction times at each stage is critical for achieving the desired chemical transformations and obtaining PBZC with the targeted properties.

### 2.3. Tailoring Porous Carbon: The Intricate Synthesis of Polybenzoxazine Aerogel Carbon (PBZGC)

The synthesis of PBZGC is a fascinating journey, meticulously crafting a porous carbon material with exceptional properties. This process, outlined in Scheme 1, can be broken down into several key stages: The first step involves crafting a critical starting material—the precursor sol. Here, a precise amount of hydrochloric acid (HCl) was dissolved in N, N-dimethylformamide (DMF), to create a controlled acidic environment. This solution was then meticulously combined with EEd-Bzo, a key building block for the desired polymer network. Additional DMF ensures thorough mixing, resulting in a homogenous precursor sol after a short stirring period at room temperature. The precursor sol was then carefully poured into molds, where a two-step thermal treatment transformed it into a gel-like state known as an alcogel. This transformation involves a meticulously controlled heating program. First, the molds are heated at a moderate temperature of 120 °C for a specific duration of 2 h. Subsequently, the temperature is elevated further to 140 °C and maintained for an extended period of 48 h. This staged heating allows for controlled polymerization and network formation within the sol. On completion of the heating process, the alcogel was meticulously cooled to room temperature. To remove the solvent trapped within the gel network, a solvent exchange-procedure was implemented. The alcogel was sequentially submerged in ethanol and water at room temperature. This process was repeated three times for each solvent, ensuring complete removal of the original DMF. Additionally, the solvent was changed every 12 h, maximizing the efficiency of the exchange process. Following solvent exchange, the delicate task of drying the alcogel commenced. Here, a supercritical CO_2_ drying technique was employed, where the alcogel was frozen in liq. N_2_ for 10 min and then dried in a freeze dryer, FDA5518 model, manufactured by Ilshin Biomass, for a period of 72 h. This method allows for the gentle removal of the remaining solvent without compromising the intricate pore structure of the gel. Next, the dried gels undergo a crucial transformation—carbonization. This process involves heating the gel to a high temperature (600 °C) under a controlled nitrogen atmosphere, following a ramp rate of 1 °C/min to ensure uniform conversion. The high temperature treatment transforms the organic polymer network into a carbon framework, retaining the desired porous structure. Further, the carbonized gel undergoes activation by intimately mixing it with potassium hydroxide (KOH) in a specific ratio (1:2), and then subjecting it to another heating program (with a ramp rate of 3 °C/min up to 800 °C and holding it at this temperature for 1 h) in a tube furnace under a nitrogen flow. The sample obtained after activation was repeatedly washed with a diluted hydrochloric acid solution (1 M HCl) followed by deionized water. This washing step removes any residual KOH and ensures the final PBZGC has a neutral pH, indicating the absence of any acidic or basic impurities. The PBZGC sample was finally dried at 110 °C for 12 h for the removal of any residual moisture. This intricate multi-step process, with its precisely controlled parameters, allows for the creation of PBZGC with specific properties tailored to various advanced material applications.

## 3. Structural Analysis of EEd-Bzo

### 3.1. FT-IR Spectroscopy

**Figure 1:**
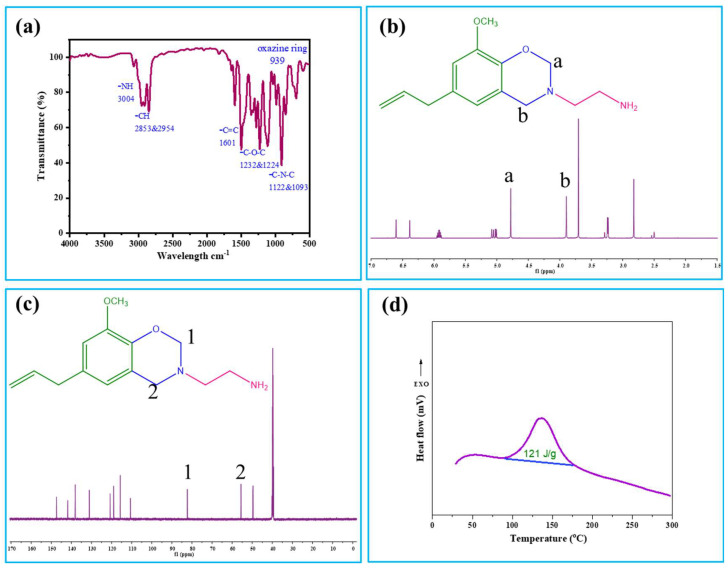
(**a**) FT-IR; (**b**) ^1^H-NMR; (**c**) ^13^C-NMR; and (**d**) DSC thermogram of EEd-Bzo monomer.

The FT-IR spectrum of the EEd-Bzo benzoxazine monomer, depicted in Figure 1, reveals several characteristic bands indicative of its molecular structure. A notable band at 939 cm^−1^ corresponds to the –CH_2_ stretching vibrations within the oxazine ring. The C–O–C asymmetric and symmetric stretching vibrations appear as distinct peaks at 1232 and 1224 cm^−1^, respectively. Additionally, the C–N–C stretching vibrations manifest as peaks at 1122 and 1093 cm^−1^. A band at 1268 cm^−1^ is attributed to the methoxy group stretching vibrations, while the tetra-substituted benzene ring gives rise to a band at 1365 cm^−1^. Aliphatic C–H stretching vibrations are identified at 2953 and 2854 cm^−1^, whereas the amine group’s –NH stretching vibrations are observed at 3004 cm^−1^. These spectral features collectively confirm the successful formation of the benzoxazine monomer [24,25].

### 3.2. NMR Spectroscopy

The structure of the benzoxazine monomer is further validated using NMR spectroscopy. Figure 1b,c illustrate the ^1^H-NMR and ^13^C-NMR spectra, respectively. The ^1^H-NMR spectrum features two singlets at 4.8 and 3.9 ppm, corresponding to the oxazine ring protons O–CH_2_–N and N–CH_2_–Ph. The amine group’s protons produce a peak at 2.8 ppm, while the methoxy group’s protons resonate at 3.7 ppm. Additionally, the methyl protons of the ethylamine group appear at 3.2 ppm, and the allyl protons are observed at 5.0 and 5.9 ppm. The aromatic protons are detected at 6.3 and 6.6 ppm. In the ^13^C-NMR spectrum, the oxazine ring carbons exhibit signals at 82.3 ppm for O–C–N and at 55.6 ppm for N–C–Ph carbons. The methylene carbons produce signals at 49.6 and 40.1 ppm, while the allyl carbons show signals at 138 and 115 ppm. The aromatic carbons resonate between 110 and 147 ppm. The peaks and signals observed in both FT-IR and NMR spectra collectively corroborate the formation of the benzoxazine ring, thereby confirming the successful synthesis of the benzoxazine monomer [24,25,26,27].

Overall, the detailed spectroscopic analysis via FT-IR and NMR provides robust evidence for the structural integrity and successful synthesis of the EEd-Bzo monomer. The presence of characteristic vibrational bands and chemical shifts specific to the oxazine ring and other functional groups confirm the anticipated molecular framework. This comprehensive spectroscopic validation underscores the reliability of the synthetic process and the structural formation of the benzoxazine monomer.

### 3.3. Unveiling a Lower-Temperature Polymerization Pathway for EEd-Bzo

Differential Scanning Calorimetric (DSC) analysis sheds light on the unique polymerization process of EEd-Bzo (Figure 1d). The presence of a distinct exothermic peak at 148 °C signifies the ring-opening polymerization of the benzoxazine unit within EEd-Bzo. This curing temperature stands out compared to traditional mono-benzoxazines like P-A (phenol-aniline), which typically require significantly higher temperatures (around 255 °C) to initiate polymerization. Therefore, the DSC analysis reveals a fascinating aspect of EEd-Bzo: its ability to polymerize at a considerably lower temperature compared to conventional mono-benzoxazines. This behavior can be primarily attributed to the –NH_2_ group, which acts as a bridge and facilitates robust crosslinking interactions with the methoxy and amine groups, thereby accelerating the polymerization process. These interactions significantly enhance the efficiency of benzoxazine ring-opening polymerization. A deeper understanding of these interactions and their impact on the polymerization behavior paves the way for the development of novel materials with precisely controlled properties [24,26].

## 4. Characterization of Carbon Structure and Graphitization

**Figure 2:**
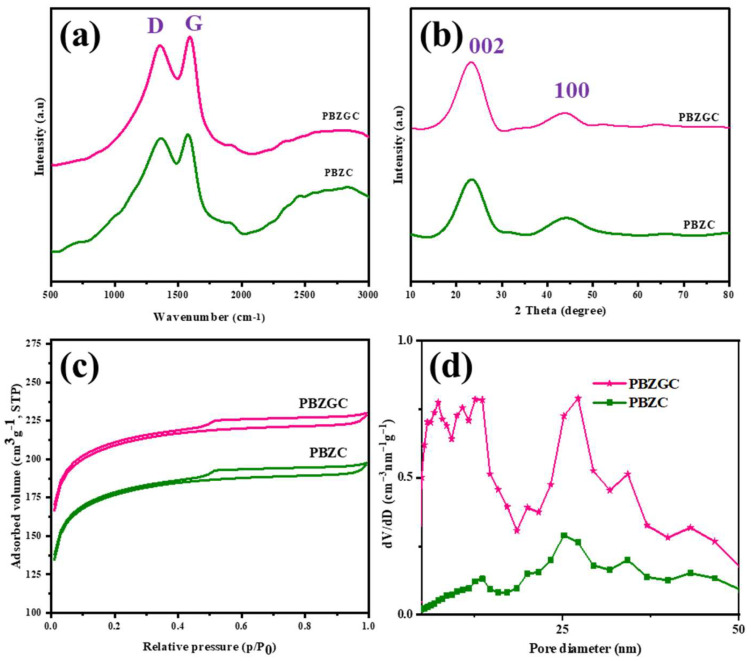
Structural characterization of the carbon materials (**a**) Raman; (**b**) XRD; (**c**) BET; and (**d**) pore-size distribution.

Raman spectroscopy and X-ray diffraction (XRD) were used to analyze the structure and graphitic properties of carbon samples. The Raman spectra showed two main peaks corresponding to disordered (D band) and graphitic (G band) carbon. The PBZC sample had a stronger D band, indicating more defects compared to PBZGC (Figure 2a). The intensity ratio of the D and G bands (I_D_/I_G_) is a measure of the graphitic quality of the carbon. The I_D_/I_G_ ratios were found to be 0.97 for PBZC and 0.84 for PBZGC. This suggests that the aerogel method and chemical activation had a minimal impact on the overall graphitic character, despite their similar chemical compositions. The lower I_D_/I_G_ ratio for PBZGC implies a higher degree of graphitization and reduced disorder, compared to PBZC. XRD analysis confirmed the presence of graphitic domains in both samples (Figure 2b). The calculated d-spacing for the (002) plane in the carbon aerogel was 0.40 nm, larger than that of traditional graphite. This increased d-spacing could potentially enhance supercapacitor performance. Raman spectroscopy and XRD provided valuable insights into the structural and graphitic properties of the carbon samples. PBZGC exhibited a higher degree of graphitization compared to PBZC, while both materials possessed graphitic domains with an expanded interlayer spacing, potentially beneficial for supercapacitors [12,16,19].

## 5. Porosity Characterization of Synthesized Carbon Materials

Nitrogen sorption analysis probed the porosity and texture of the synthesized samples. Brunauer–Emmett–Teller (BET) theory and non-localized density functional theory (NLDFT) revealed specific surface area and pore-size distribution (PSD). Figure 2c showcases the nitrogen adsorption behavior. Interestingly, the isotherms exhibited a combination of type I and IV characteristics. The sharp rise at low relative pressures (P/P_0_) suggests the presence of micropores in PBZGC, particularly evident compared to PBZC. Furthermore, both samples displayed hysteresis loops throughout the P/P_0_ range, indicative of mesopores with consistent size. Figure 2d confirms the presence of mesopores (20–50 Å) in both samples. Notably, PBZC (calcination derived) possessed a broader mesopore distribution compared to the narrower and higher volume distribution observed in PBZGC (aerogel derived). BET analysis yielded specific surface areas of 576 m^2^ g^−1^ for PBZC and 843 m^2^ g^−1^ for PBZGC. The combination of micropores, high surface area, and well-defined mesopores makes PBZC and PBZGC most suitable to be used as electrode materials for supercapacitor applications. This optimized microstructure facilitates efficient electrochemical processes—a critical aspect for next-generation energy storage devices [28,29].

## 6. Unveiling the Chemical Landscape: XPS Analysis of Nitrogen-Doped Carbon Aerogels

**Figure 3:**
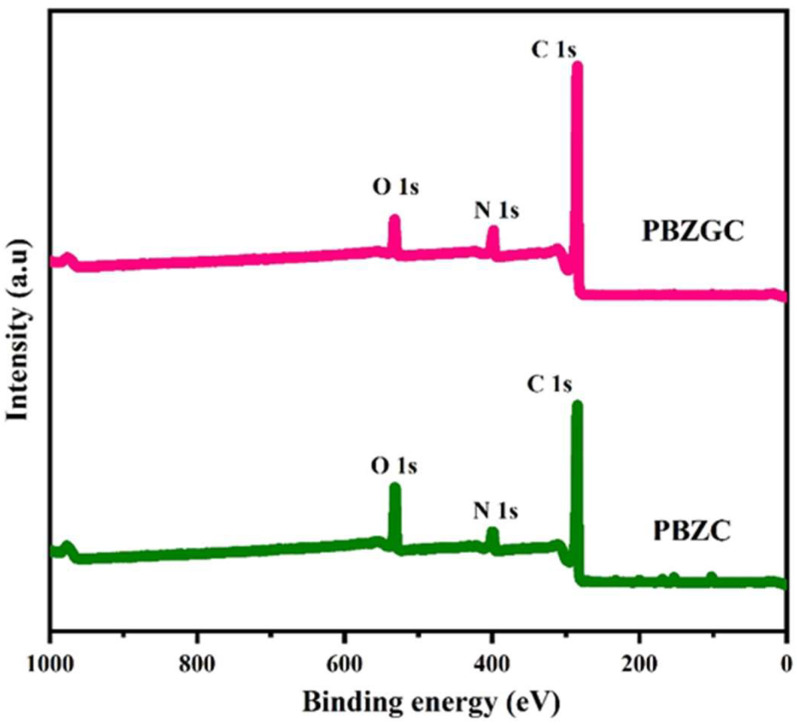
XPS survey spectra of PBZC and PBZGC.

**Figure 4:**
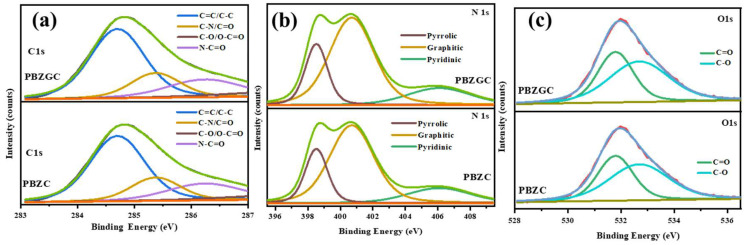
De-convoluted XPS spectra of PBZC and PBZGC (**a**) C 1s; (**b**) N 1s; and (**c**) O 1s.

X-ray Photoelectron Spectroscopy (XPS) served as a powerful tool to decipher the interplay between nitrogen and oxygen species on the surfaces of the fabricated carbon aerogels. Figure 3 and Figure 4 showcase the XPS spectra of the synthesized carbon samples—PBZC and PBZGC—revealing distinct peaks for carbon (C 1s), nitrogen (N 1s), and oxygen (O 1s). These signatures strongly confirm the successful incorporation of nitrogen and oxygen into the carbon frameworks, as expected when using benzoxazine monomers as the starting material. Notably, the XPS analysis also verifies the absence of any contaminants in the self-doped carbon materials. Figure 3 depicts a broad XPS survey for both samples, highlighting the presence of carbon, nitrogen, and oxygen at characteristic binding energies of approximately 285.2, 400.1, and 532.3 eV, respectively. A deeper dive into the specific chemical environments is presented in Figure 4 through a detailed examination of the C 1s, N 1s, and O 1s signals [30,31,32].

Deconvolution of the C 1s peak (Figure 4a) unveiled four distinct contributions: C=C/C–C hydrocarbon chains (284.8 eV), C–N bonds (285.6 eV), O–C=O/C=N/C–OH groups (286.5 eV), and carbon atoms bonded with oxygen and nitrogen in HN–C=O groups (288.8 eV). This detailed breakdown provides valuable insights into the various carbon bonding configurations within the aerogel structure. The N 1s region (Figure 4b) revealed three distinct peaks corresponding to different types of nitrogen functionalities: pyrrolic N (398.4 eV), graphitic N (400.6 eV), and pyridine N-oxide (406.3 eV). This variety of nitrogen incorporation suggests a complex yet potentially beneficial chemical environment for specific applications. The O 1s spectrum (Figure 4c) further enriches the picture by displaying four peaks at binding energies of 531.5, 533.1, 533.6, and 537.2 eV, respectively, attributed to hydroxyl (C–OH), epoxy (C–O–C), carbonyl (C=O)/carboxyl (COO), and chemisorbed oxygen or water groups [33,34,35]. This confirms the presence of oxygen-containing functional groups and potentially entrapped water molecules within the carbon matrix. Similar observations could be observed for PBZGC with a slight shift in the deconvoluted peaks of C 1s, N 1s, and O 1s. This further underscores the presence of diverse nitrogen bonding-configurations within the PBZGC. In addition, the XPS analysis serves as a compelling validation of the successful nitrogen doping within the synthesized carbon materials. The observed higher nitrogen content compared to reference materials like APFC-N suggests a promising avenue for enhanced performance in applications like supercapacitors. This comprehensive XPS characterization offers valuable insights into the chemical states and interactions within the activated and aerogel-based carbons, paving the way for their exploration in energy storage technologies.

## 7. Contrasting Morphologies of the Carbon Materials

**Figure 5:**
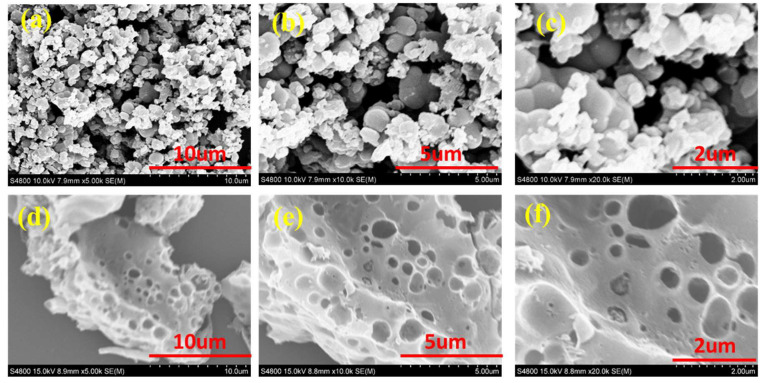
FE-SEM images of (**a**–**c**) PBZC; and (**d**–**f**) PBZGC at different magnifications.

Scanning electron microscopy (SEM) revealed distinct textural differences between PBZC and PBZGC, impacting their potential for supercapacitor applications. PBZC exhibited an irregular microparticle structure with particle sizes ranging from hundreds of nanometers to a few micrometers. These particles clumped together to form large, porous clusters with a spreading of flaky particles in some regions, and numerous balls in another region (Figure 5a–c). Notably, PBZC lacks visible pores, displaying a rough and continuous surface. This absence of porosity translated into a limited surface area, potentially hindering its supercapacitor performance. In contrast, PBZGC, derived from activated carbon aerogel, boasted a dramatically transformed surface morphology. SEM images unveiled a network of micropores and mesopores scattered across the PBZGC surface, with minimal presence of larger macropores. These macropores, roughly around 0.5 mm in diameter (Figure 5d–f), likely arose from the expulsion of residual solvent during gelation. This extensive network of pores is highly beneficial for supercapacitors as it significantly expands the surface area, providing more sites for interaction with electrolyte ions and, consequently, enhancing capacitance.

**Figure 6:**
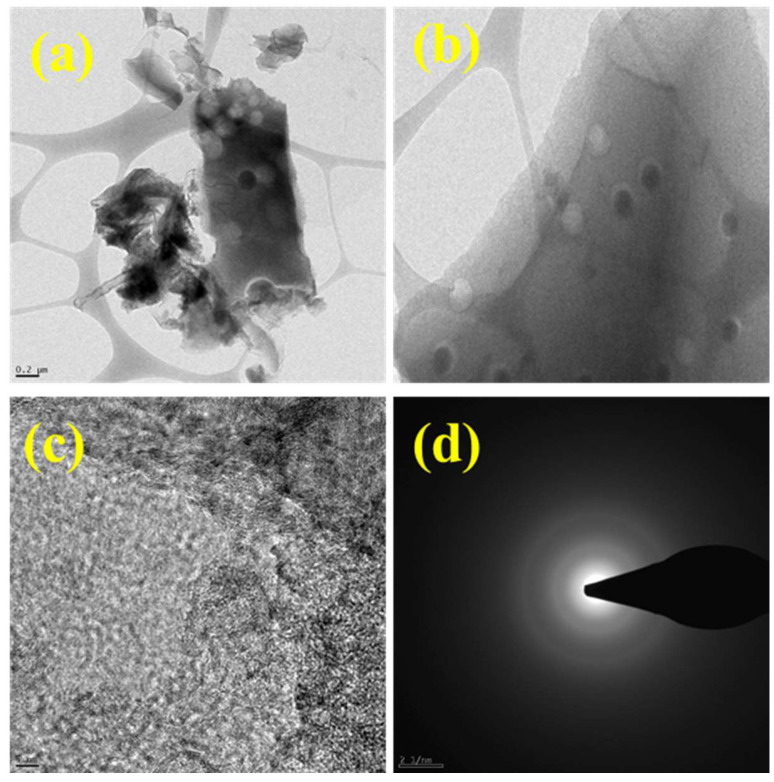
FE-TEM images of (**a**–**c**) PBZGC at different magnifications; and (**d**) SAED pattern.

Transmission electron microscopy (TEM) offered a closer look at PBZGC’s internal structure. The high-resolution images (Figure 6a–d) reveal an open-pore network, a crucial characteristic for optimal supercapacitor performance. This nano-architecture offers several advantages: it shortens the diffusion pathways for ions, enabling their rapid movement within the electrode, and it guarantees a continuous path for electrons, thereby improving electrical conductivity. Additionally, PBZGC exhibited a denser network of pores with a more ordered arrangement, suggesting the presence of sp^2^-bonded carbon. This is particularly advantageous because sp^2^-bonded carbon is renowned for its exceptional electrical conductivity.

In essence, SEM and TEM analyses unveiled significant discrepancies between PBZC and PBZGC. PBZC’s limited porosity and irregular structure suggest it may be less suitable for supercapacitor applications. Conversely, PBZGC’s highly porous and well-ordered structure, coupled with the presence of sp^2^-bonded carbon, implies superior performance due to its increased surface area and enhanced electrical conductivity. These morphological characteristics are paramount for optimizing the efficiency and performance of supercapacitor electrodes.

## 8. Electrochemical Studies

### Nitrogen-Rich Porous Carbons for Supercapacitors: PBZC and PBZGC

**Figure 7:**
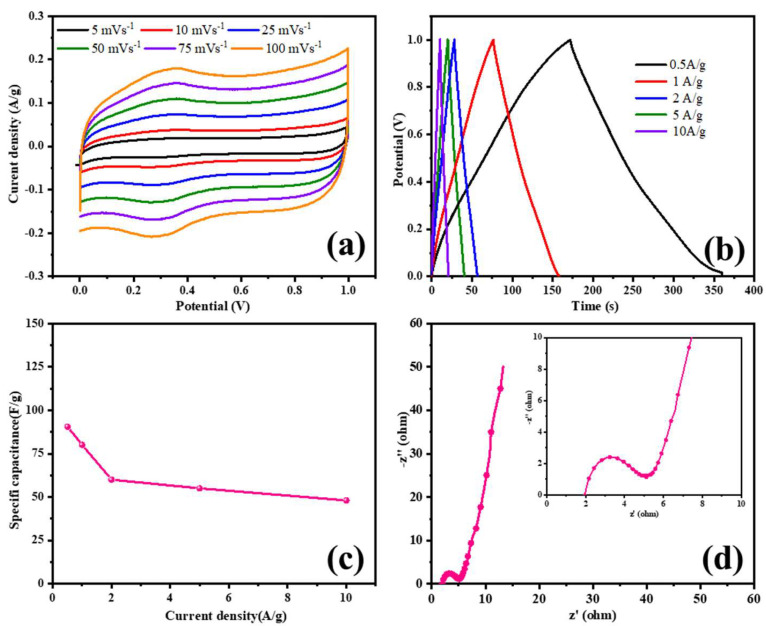
Electrochemical characterizations of PBZC: (**a**) CV graphs; (**b**) GCD curves; (**c**) specific capacitance; and (**d**) EIS spectrum.

**Figure 8:**
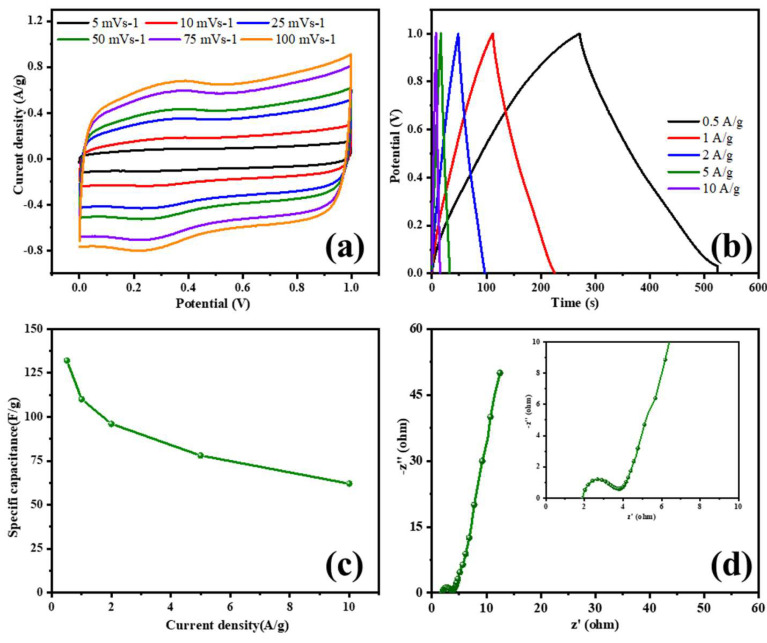
Electrochemical characterizations of PBZGC: (**a**) CV graphs; (**b**) GCD curves; (**c**) specific capacitance; and (**d**) EIS spectrum.

The prepared materials—PBZC and PBZGC—are nitrogen-rich porous carbons with unique microstructures, which makes them ideal to be used as supercapacitor electrodes. A comprehensive electrochemical analysis using a three-electrode system with sulfuric acid as an electrolyte was conducted to assess their potential. Cyclic voltammetry (CV) is a key technique used to examine the capacitive properties of these electrodes. As shown in Figure 7a and Figure 8a, the CV curves for both PBZC and PBZGC exhibit a near-rectangular shape, even at high scan rates, indicating excellent capacitive behavior. Notably, the larger CV curve area for PBZGC suggests superior charge storage and release capabilities, compared to PBZC. Further insights into charge storage and delivery were obtained through galvanostatic charge–discharge (GCD) measurements. The GCD curves for PBZC and PBZGC at different current densities are displayed in Figure 7b and Figure 8b, respectively. They show near-triangular shaped GCD curves, with minimal voltage drops during charge and discharge, indicating efficient charge storage. Slight deviations from ideal triangular shapes suggest some pseudocapacitive behavior, likely due to the presence of nitrogen atoms confirmed by X-ray photoelectron spectroscopy (XPS). These nitrogen functionalities enhance pseudocapacitance by creating an electrochemically active interface with the electrolyte ions [36,37,38].

Specific capacitance, a crucial performance metric for supercapacitors, is influenced by current density. As evident in Figure 7c and Figure 8c, the specific capacitance (C_s_) at a current density of 0.5 A g^−1^ was found to be 90.5 F g^−1^ for PBZC and 132 F g^−1^ for PBZGC. Moreover, the specific capacitance of both the electrodes decreases with increasing current density. This decrease highlights the limitations of the electrode materials at high charging and discharging rates. The electrochemical analysis revealed superior capacitive behavior and charge storage efficiency for PBZGC, compared to PBZC. Nitrogen functionalities play a significant role in enhancing pseudocapacitance, but further optimization is required to improve the rate capabilities of these materials for practical supercapacitor applications.

Electrochemical impedance spectroscopy (EIS) stands as a crucial tool in investigating the complexities of electrode–electrolyte interfaces within supercapacitors, offering profound insights into their operational dynamics. This technique delves into key parameters such as interfacial dynamics, diffusion kinetics, electronic conductivity, and charge-transfer resistance (R_ct_), all pivotal in determining the overall performance of these energy storage devices. Figure 7d and Figure 8d present Nyquist plots depicting the impedance characteristics of PBZC and PBZGC electrodes, respectively, integral to their electrochemical characterization. These plots are instrumental in discerning critical parameters: the intercept on the real axis signifies the solution resistance (R_s_), while the semicircle diameter reflects R_ct_. Notably, PBZGC exhibits a significantly lower R_ct_ of 2.1 Ω compared to PBZC 3.7 Ω, indicating superior electronic conductivity crucial for enhancing its rate capability, a fundamental attribute for high-performance supercapacitors [39,40,41,42].

**Figure 9:**
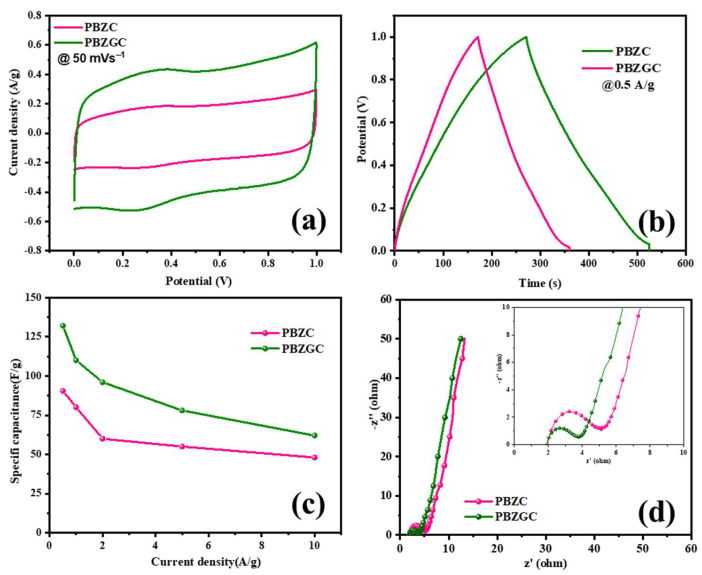
Comparison of electrochemical results of PBZC and PBZGC: (**a**) CV graphs; (**b**) GCD curves; (**c**) specific capacitance; and (**d**) EIS spectrum.

The diminished performance observed in PBZC is attributed to its inadequate porosity, resulting in reduced surface area and uneven distribution of nitrogen atoms within the material. In contrast, PBZGC, derived from biomass and enriched with nitrogen content, emerges as a promising alternative electrode material. Its nearly rectangular cyclic voltammetry (CV) curves, triangular galvanostatic charge–discharge (GCD) profiles, and lower R_s_ and R_ct_ values underscore its exceptional capacitive behavior and robust rate capability. Further insights from Figure 9a–d provide a detailed comparative analysis of the electrode materials. The CV curve of PBZGC at 50 mV s^−1^ exhibits a significantly larger area compared to PBZC, indicative of higher capacitance. Similarly, GCD curves at 0.5 A g^−1^ illustrate PBZGC’s longer discharge time, highlighting its superior charge storage and release capabilities. The superior performance of PBZGC can be attributed to its unique microporous structure, complemented by micro-, meso-, and macropores. Micropores play a crucial role by offering a large surface area essential for efficient ion adsorption, thereby contributing to pseudocapacitance. Meso- and macropores facilitate uniform infiltration of electrolyte throughout the electrode, maximizing the accessible surface area for enhanced ionic interactions. The synergy among these pore sizes and their distribution is pivotal in achieving high capacitance and superior electrochemical performance [43,44,45].

**Figure 10:**
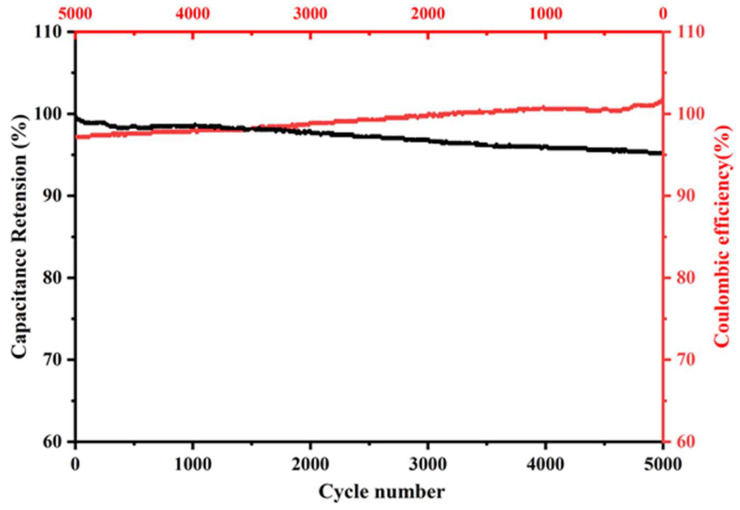
Cyclic stability of PBZGC showing capacitance retention and coulombic efficiency for 5000 cycles.

The findings presented in Figure 9c demonstrate a substantial enhancement in specific capacitance (C_s_) when incorporating a gel network into the PBZGC electrode. At a current density of 0.5 A g^−1^, the PBZC electrode achieves a Cs of 90.5 F g^−1^, while the PBZGC electrode remarkably achieves 132 F g^−1^. This significant improvement is directly attributed to the optimized porous structure of PBZGC. The interconnected network of pores facilitates efficient electrolyte penetration, thereby maximizing the electrode’s surface area available for ion interaction. This structural advantage fulfills a critical requirement for achieving high capacitance, firmly establishing PBZGC as a highly promising material for supercapacitor applications. To further evaluate the practical feasibility of PBZGC electrodes, their cycling stability was assessed through a Galvanostatic Charge–Discharge (GCD) study over 5000 cycles (Figure 10). Notably, the C_s_ of the PBZGC electrode demonstrated exceptional stability throughout the extensive cycling test at a current density of 0.5 A g^−1^. Despite a slight decrease in specific capacitance from 132 to 124 F g^−1^ after 5000 cycles, the capacitance retention ratio remained impressive at 94%. This outstanding stability underscores the long-term durability of PBZGC electrodes, demonstrating their suitability for real-world applications in supercapacitor devices.

## 9. Conclusions

We developed a new method to create activated porous carbons containing heteroatoms (PBZC and PBZGC) from polybenzoxazine. This innovative approach avoids the use of complex templates and proceeds through a simpler and more cost-effective process. The key lies in using polybenzoxazine as a precursor. This allows the creation of a carbon aerogel using eco-friendly dimethyl sulfoxide as a solvent. The resulting aerogel has an optimized pore-size distribution, making it ideal for electrochemical applications, especially as supercapacitor electrodes. This method not only simplifies production but also enhances the functionality of the carbon aerogel. Notably, the research highlights the advantages of the biomass-derived PBZC electrode compared to PBZGC. The hierarchical pore structure and the presence of heteroatoms in PBZGC contribute to its superior performance. The optimized pores allow for better electrolyte penetration, and the high surface area (843 m^2^ g^−1^) of PBZGC translates to significant improvements in capacitance and cycling stability. These features make PBZGC a promising candidate for high-performance supercapacitors. The well-designed structure, featuring a mix of micropores, mesopores, and macropores facilitates efficient ion transport and storage. This, combined with the heteroatoms, boosts capacitance (132 F g^−1^) and ensures remarkable stability (94% capacitance retention) over extended use. These characteristics are crucial for practical supercapacitor applications. This research demonstrates the potential of bio-based materials for energy storage technologies. By using sustainable resources like benzoxazine, carbon materials containing hetero-atoms were produced, which enhances the capacitance by producing pseudocapacitance in addition to EDLC. This work paves the way for next-generation supercapacitors, while highlighting the potential of eco-friendly approaches in materials science. Further exploration and optimization of these materials hold promise for continued advancements in sustainable energy storage solutions.

## Data Availability

Data are contained within the article.

## References

[1] Biesinger M.C., Payne B.P., Grosvenor A.P., Lau L.W.M., Gerson A.R., Smart R.S.C. (2011). Resolving Surface Chemical States in XPS Analysis of First Row Transition Metals, Oxides and Hydroxides: Cr, Mn, Fe, Co and Ni. Appl. Surf. Sci..

[2] Rittidech A., Portia L., Bongkarn T. (2006). The Relationship between Microstructure and Mechanical Properties of Al_2_O_3_-MgO Ceramics. Mater. Sci. Eng. A.

[3] Riu D.H., Kong Y.M., Kim H.E. (2000). Effect of Cr_2_O_3_ Addition on Microstructural Evolution and Mechanical Properties of Al_2_O_3_. J. Eur. Ceram. Soc..

[4] Ali N., Bashir S., Begum N., Rafique M.S., Husinsky W. (2017). Effect of Liquid Environment on the Titanium Surface Modification by Laser Ablation. Appl. Surf. Sci..

[5] Vlasova M., Aguilar P.A.M., Kakazey M., Reséndiz-González M.C., Bykov A., Ragulya A., Tomila T. (2007). Modification of a SiC-Cr_5_Si_3_ Ceramic Surface by Laser Irradiation. Ceram. Int..

[6] Sartinska L.L., Barchikovski S., Wagenda N., Rud’ B.M., Timofeeva I.I. (2007). Laser Induced Modification of Surface Structures. Appl. Surf. Sci..

[7] Stolz B., Backes G., Gillner A., Kreutz E.W. (1997). Selective Surface Modification of Ceramics with Laser Radiation. Appl. Surf. Sci..

[8] Ouraipryvan P., Sreethawong T., Chavadej S. (2009). Synthesis of Crystalline MgO Nanoparticle with Mesoporous-Assembled Structure via a Surfactant-Modified Sol-Gel Process. Mater. Lett..

[9] Xu X., Hu S., Pan Q., Huang Y., Zhang J., Chen Y., Wang H., Zheng F., Li Q. (2024). Enhancing Structure Stability by Mg/Cr Co-Doped for High-Voltage Sodium-Ion Batteries. Small.

[10] Zhang H., Wang M., Jia Q., Zhang Z., Lei L., Chen L. (2024). Corrosion Mechanism of Reactive MgO-Bonded Cr_2_O_3_-Bearing Castables in CaO–Al_2_O_3_–Fe_2_O_3_–SiO_2_-Based Steel-Making Slag. J. Am. Ceram. Soc..

[11] Pourdelan H., Alavi S.M., Rezaei M., Akbari E., Klyamkin S. (2023). Production of Pure Hydrogen through Thermocatalytic Methane Decomposition Using NiO-MgO Catalysts Promoted by Chromium and Copper Prepared via Mechanochemical Method. Int. J. Energy Res..

[12] Hu Y., Guo Y., Sun J., Li H., Liu W. (2019). Progress in MgO Sorbents for Cyclic CO_2_ Capture: A Comprehensive Review. J. Mater. Chem. A.

[13] Choi D., Park Y. (2022). Structural Modification of Salt-Promoted MgO Sorbents for Intermediate Temperature CO_2_ Capture. Nanoscale Adv..

[14] Li S., Zhan S., Sun J., Yao L., Zhu J., Feng J., Xiong Y., Tian S. (2021). Enhanced Ozonation of Pollutants by MgO Nanoclusters/Sewage Sludge-Derived Hierarchical Porous Carbon: Experimental and Theoretical Study. Environ. Sci. Nano.

[15] Mori K., Fujita T., Yamashita H. (2023). Boosting the Activity of PdAg Alloy Nanoparticles during H_2_ Production from Formic Acid Induced by CrOx as an Inorganic Interface Modifier. EES Catal..

[16] Zhou J., Xiao Y., Liu S., Li Z., Liu X., Zhang S., Li Z. (2024). Boric Acid-Regulated Gelation and Ethanol-Assisted Preparation of Polybenzoxazine Aerogels. Chem. Eng. J..

[17] Mansoor M.A., Munawar K., Naeem R., Sarih N.M., Asghar M.A., Haider A., Zubir M.N.M., Zaharinie T. (2023). Aerosol-Assisted Facile Fabrication of Bimetallic Cr_2_O_3_-Mn_2_O_3_ Thin Films for Photoelectrochemical Water Splitting. New J. Chem..

[18] Bhateja Y., Ghosh R., Sponer J., Majumdar S., Cassone G. (2022). A Cr_2_O_3_-Doped Graphene Sensor for Early Diagnosis of Liver Cirrhosis: A First-Principles Study. Phys. Chem. Chem. Phys..

[19] Jan F., Zhi S., Sun X.Y., Li B. (2024). Enhancing Catalytic Activity of Cr_2_O_3_ in CO_2_-Assisted Propane Dehydrogenation with Effective Dopant Engineering: A DFT-Based Microkinetic Simulation. Phys. Chem. Chem. Phys..

[20] Ding J., Ming J., Lu D., Wu W., Liu M., Zhao X., Li C., Yang M., Fang P. (2017). Study of the Enhanced Visible-Light-Sensitive Photocatalytic Activity of Cr_2_O_3_-Loaded Titanate Nanosheets for Cr(VI) Degradation and H_2_ Generation. Catal. Sci. Technol..

[21] Herdiech M.W., Zhu X., Morales-Acosta M.D., Walker F.J., Altman E.I. (2015). The Modification of Ferroelectric LiNbO_3_(0001) Surfaces Using Chromium Oxide Thin Films. Phys. Chem. Chem. Phys..

[22] Edison T.N.J.I., Atchudan R., Sethuraman M.G., Lee Y.R. (2016). Supercapacitor performance of carbon supported Co_3_O_4_ nanoparticles synthesized using Terminalia chebula fruit. J. Taiwan Inst. Chem. Eng..

[23] Periyasamy T., Asrafali S.P., Lee J. (2024). High-Performance Supercapacitor Electrodes from Fully Biomass-Based Polybenzoxazine Aerogels with Porous Carbon Structure. Gels.

[24] Thirukumaran P., Parveen A.S., Sarojadevi M. (2014). Synthesis and Copolymerization of Fully Biobased Benzoxazines from Renewable Resources. ACS Sustain. Chem. Eng..

[25] Thirukumaran P., Atchudan R., Parveen A.S., Lee Y.R., Kim S.-C. (2018). Polybenzoxazine originated N-doped mesoporous carbon ropes as an electrode material for high-performance supercapacitors. J. Alloys Compd..

[26] Thirukumaran P., Atchudan R., Balasubramanian R., Parveen A.S., Kim S.-C. (2018). Direct synthesis of nitrogen-rich carbon sheets via polybenzoxazine as highly active electrocatalyst for water splitting. Int. J. Hydrogen Energy.

[27] Periyasamy T., Asrafali S.P., Muthusamy S. (2014). New benzoxazines containing polyhedral oligomeric silsesquioxane from eugenol, guaiacol and vanillin. New J. Chem..

[28] Atchudan R., Edison T.N.J.I., Perumal S., Lee Y.R. (2017). Green synthesis of nitrogen-doped graphitic carbon sheets with use of Prunus persica for supercapacitor applications. Appl. Surf. Sci..

[29] Atchudan R., Perumal S., Karthikeyan D., Pandurangan A., Lee Y.R. (2015). Synthesis and characterization of graphitic mesoporous carbon using metal–metal oxide by chemical vapor deposition method. Microporous Mesoporous Mater..

[30] Lamberti C., Zecchina A., Groppo E., Bordiga S. (2010). Probing the Surfaces of Heterogeneous Catalysts by in Situ IR Spectroscopy. Chem. Soc. Rev..

[31] Yang L., Wang Y., Sui C., Liu Z., Liu Y., Li Y., Bai J., Liu F., Lu G. (2022). Highly Selective and Humidity-Resistant Triethylamine Sensors Based on Pt and Cr_2_O_3_Nanoparticles. ACS Appl. Nano Mater..

[32] Li S., Chen W., Huang X., Ding L., Ren Y., Xu M., Zhu J., Miao Z., Liu H. (2024). Enabling Wasted A4 Papers as a Promising Carbon Source to Construct Partially Graphitic Hierarchical Porous carbon for High-Performance Aqueous Zn-Ion Storage. ACS Appl. Mater. Interfaces.

[33] Song B.Y., Zhang X.F., Huang J., Cheng X.L., Deng Z.P., Xu Y.M., Huo L.H., Gao S. (2022). Porous Cr_2_O_3_ Architecture Assembled by Nano-Sized Cylinders/ Ellipsoids for Enhanced Sensing to Trace H_2_S Gas. ACS Appl. Mater. Interfaces.

[34] Simeonidis K., Kalaitzidou K., Asimakidou T., Martinez-Boubeta C., Makridis A., Haeussler A., Vourlias G., Balcells L. (2023). Tin Oxide Nanoparticles via Solar Vapor Deposition for Hexavalent Chromium Remediation. ACS Appl. Nano Mater..

[35] Lodesani A., Picone A., Brambilla A., Giannotti D., Jagadeesh M.S., Calloni A., Bussetti G., Berti G., Zani M., Finazzi M. (2019). Graphene as an Ideal Buffer Layer for the Growth of High-Quality Ultrathin Cr_2_O_3_ Layers on Ni(111). ACS Nano.

[36] Kurashige W., Kumazawa R., Ishii D., Hayashi R., Niihori Y., Hossain S., Nair L.V., Takayama T., Iwase A., Yamazoe S. (2018). Au_25_-Loaded BaLa_4_Ti_4_O_15_ Water-Splitting Photocatalyst with Enhanced Activity and Durability Produced Using New Chromium Oxide Shell Formation Method. J. Phys. Chem. C.

[37] Chanuta N., Stefaniuk D., Weaver J.C., Zhu Y., Yang S.H., Masic A., Ulm F.J. (2023). Carbon–cement supercapacitors as a scalable bulk energy storage solution. Proc. Natl. Acad. Sci. USA.

[38] Aouani H., Wenger J., Gérard D., Rigneault H., Devaux E., Ebbesen T.W., Mahdavi F., Xu T., Blair S. (2009). Crucial Role of the Adhesion Layer on the Plasmonic Fluorescence Enhancement. ACS Nano.

[39] Zuñiga-Ibarra V.A., Shaji S., Krishnan B., Johny J., Sharma Kanakkillam S., Avellaneda D.A., Martinez J.A.A., Roy T.K.D., Ramos-Delgado N.A. (2019). Synthesis and Characterization of Black TiO_2_ Nanoparticles by Pulsed Laser Irradiation in Liquid. Appl. Surf. Sci..

[40] García-Quiñonez L.V., Mendivil-Palma M.I., Roy T.K.D., Castillo-Rodríguez G.A., Gómez-Rodríguez C., Fernández-González D., Shaji S. (2020). Effects of Irradiation Energy and Nanoparticle Concentrations on the Structure and Morphology of Laser Sintered Magnesia with Alumina and Iron Oxide Nanoparticles. Ceram. Int..

[41] Guzmán K.d.C.M., Shaji S., Das Roy T.K., Krishnan B., Avellaneda D.A., Aguilar Martinez J.A., Valdes J.J.R. (2021). Surface Modification of Sintered Magnesium Oxide (MgO) with Chromium Oxide (Cr_2_O_3_) by Pulsed Laser Irradiation in Air and Liquids. Ceram. Int..

[42] Zhang Y., Zhou C., Yan X., Cao Y., Gao H., Luo H., Gao K., Xue S., Jing X. (2023). Recent Advances and Perspectives on Graphene-Based Gels for Superior Flexible All-Solid-State Supercapacitors. J. Power Sources.

[43] Salleh N.A., Kheawhom S., Hamid N.A.A., Rahiman W., Mohamad A.A. (2023). Electrode Polymer Binders for Supercapacitor Applications: A review. J. Mater. Res. Technol..

[44] Sahoo S., Sahoo G., Jeong S.M., Rout C.S. (2022). A review on supercapacitors based on plasma enhanced chemical vapor deposited vertical graphene arrays. J. Energy Storage.

[45] Manickam M., Achini S., Jonathan W., Rob A., Pragati A.S., Katsuhiko A., Lok Kumar S. (2024). Phosphorous—Containing Activated Carbon Derived From Natural Honeydew Peel Powers Aqueous Supercapacitors. Chem. Asian J..

